# Infant gut microbiome reprogramming following introduction of solid foods (weaning)

**DOI:** 10.1080/19490976.2025.2571428

**Published:** 2025-11-09

**Authors:** Mengfan Ding, R. Paul Ross, Eugene Dempsey, Bowen Li, Catherine Stanton

**Affiliations:** a APC Microbiome Ireland, University College Cork, Cork, Ireland; b Teagasc Food Research Centre, Moorepark, Fermoy, Ireland; c Department of Paediatrics and Child Health, University College Cork, Cork, Ireland; d INFANT Research Centre, University College Cork, Ireland; e College of Food Science, Southwest University, Chongqing, China

**Keywords:** Weaning, gut microbiome, composition, function

## Abstract

The infant gut microbiome undergoes a crucial transformation when solid foods enter the diet during weaning. This introduction normally happens at about six months post-birth and leads to major shifts in the gut microbiome. Many of the changes that occur during this period are known to persist into adulthood. While many perinatal factors, including gestational age, delivery mode, feeding choices, and antibiotic exposure, strongly influence microbiome composition and functional trajectories, the effects of weaning, in particular, have received far less attention. This review examines the response of the microbiome ecosystem when the diet is radically altered through the introduction of solid foods during the weaning phase. This response involves major reshaping of anabolic and catabolic functioning, along with changes in bacterial taxa and increased diversity. The information presented in this review aims to fill existing knowledge gaps while advancing our comprehension of how the infant diet shapes gut microbiome development through childhood.

## Introduction

The gut microbiome plays an essential role in maintaining human health throughout the entire lifespan.^1^ The gut microbiota begins its colonization process at birth and experiences significant transformations until the child reaches 3 y of age.^2^ The structure of the infant gut microbiome is influenced by factors such as delivery mode, feeding type, and maternal diet for breastfed infants, gestational age at birth, antibiotic treatments, and environmental factors (i.e. living conditions).^3^^,^^4^ The infant gut microbiome thus undergoes dramatic shifts in composition, developing from an unstable neonatal microbiome to a more stable form towards an adult-like microbiome during the first year.^5^ In contrast, adult-like gut microbiomes are stable, diverse, and functionally mature microbial communities that form after infancy, typically post-weaning. They feature a shift from *Bifidobacterium* dominance to genera such as *Bacteroides*, *Clostridium*, and *Faecalibacterium*, along with a high capacity for metabolic function.^6^

Weaning refers to the dietary shift from a milk-only diet to one that includes solid foods and other fluids,^7^ which generally occurs at around the age of six months, as recommended by the World Health Organization (WHO).^8^ This transition depends on the introduction of non-dairy (as well as dairy) foods that compensate for variations in the consumption of macronutrients (proteins, fats, carbohydrates, and fibers).^3^^,^^9-14^ Before the introduction of solid foods, the infant gut is typically dominated by *Bifidobacterium*, though its abundance varies considerably across individuals and cohorts (ranging from 0.001% to 91%).^15-18^ For instance, a longitudinal study of 25 healthy, breast- or mixed-fed infants in Copenhagen reported that *Bifidobacterium* accounted for 64.3% of the gut microbiota based on 16S rDNA sequencing.^19^ Another study of 94 mother–infant pairs showed that *Bifidobacterium* represented ~50% of the microbiota at 2 weeks of age, increasing to ~75% by 6 months in breast-fed infants.^20^ However, *Bifidobacterium* is not universally present. A large-scale study involving 418 U.S. infants across 48 states revealed that 25% had no detectable *Bifidobacterium* using shotgun metagenomics, even among vaginally delivered, breastfed infants.^21^ High-throughput sequencing technologies such as Illumina have improved resolution; however, detection remains sensitive to pipeline choices – including the use of 16S rRNA vs. shotgun sequencing, primer and region selection, sequencing depth, and bioinformatic cutoffs – which may contribute to inconsistent detection of *Bifidobacterium* across studies. Following weaning, the microbiome shifts to become more similar to the adult microbiome composition.^3^^,^^22^ The introduction of solid foods induces fluctuations in the gut microbiome by providing diverse substrates,^23^ which further affect its functional composition. This “optimal” timing (weaning time) opens a window for infants to gain new microbial species and functions, which may be linked to the occurrence of disease later in life.^24^^,^^25^

The development of the infant gut microbiome during breast or formula feeding has been documented previously.^26^^,^^27^ However, few studies have examined how the introduction of supplemental foods affects the composition and function of the gut microbiome. Therefore, the aim of this review is to summarize the dynamics of infant gut microbiome composition and function to evaluate the changes induced by the introduction of complementary foods during weaning in relation to timing and dietary influences. Ultimately, these findings could help improve infant health outcomes by fostering better nutrition and microbiome development.

### Microbiota composition of infant gut after weaning

The period between 6 and 12 months represents a significant transitional stage in infant nutrition, with the introduction of supplemental foods and a progressive decline in exclusive breastfeeding. Several studies have found that this 6- to 12-month period is associated with the most extensive changes in gut microbial composition, diversity, and functional potential. While microbiota modifications persist, they are less dramatic after 12 months of age.^28^^,^^29^ As a result, using two independent age brackets, i.e. 6−12 months and >12 months, allows us to distinguish between the microbiota's dynamic establishing phase and the post-weaning maturity period, respectively. We then discuss the microbiota composition of the infant gut after weaning in these two stages.

### Microbiota composition of infant gut from 6 to 12 months

**Figure 1.**
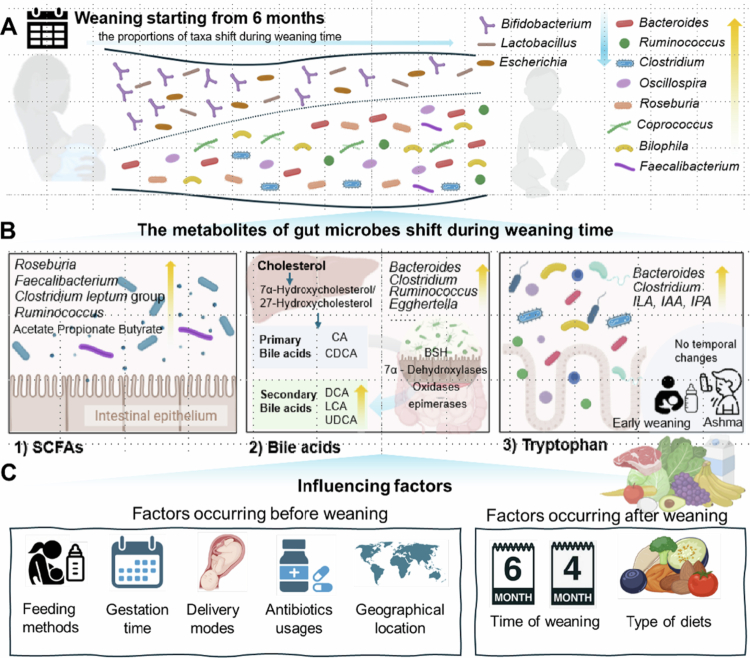
The infant gut microbiome and metabolites change during weaning and the factors influencing microbial composition in infant gut after weaning. (A) The composition of the infant gut microbiome shifts from a *Bifidobacterium*-dominant type to a more diverse microbial community after weaning. Typically, infants are weaned at approximately 6 months of age, a period when their diet begins to include solid foods alongside breast milk or formula. (B) The levels of metabolites (SCFAs, bile acids, and tryptophan) produced by the gut microbiome change during weaning, and early or late weaning may cause dysbiosis of the metabolites. (C) Multiple factors have been shown to influence the gut microbiome, including feeding method, gestation time, delivery mode, antibiotic usage, geographical location, time of weaning and type of diet. CA: Cholic acid. CDCA: Chenodeoxycholic acid. DCA: Deoxycholic Acid. LCA: Lithocholic acid. UDCA: Ursodeoxycholic acid. ILA: Indole-3-lactic acid. IAA: Indole-3-acetic acid. IPA: Indole−3-propionic acid.

The introduction of solid foods to the infant diet during the weaning phase is associated with a rapid shift in the gut microbiota composition and function (Table 1).^30^ This shift includes a sharp decrease in human milk oligosaccharide (HMO)-utilizing species and an enrichment of *Bacteroides*, *Bilophila*, *Roseburia*, *Clostridium*, and *Anaerostipes* (Figure 1A).^5^^,^^31^ This may be explained by the exposure to many non-digestible plant carbohydrates, which subsequently reach the colon and affect microbiota colonization by creating novel substrates for the survival and dominance of species that are not supported by breast milk or infant formula.^32^ Overall, the process of weaning leads to changes in alpha diversity (Shannon index) and stabilization of the infant gut microbiota.^33^^,^^34^ Increased microbial diversity is linked to the gut microbiome's capacity to adapt to diverse foods, which may explain why the introduction of solid foods marks a critical point in the gut microbiome development toward becoming more adult-like in composition.^35^

**Table 1.** The microbial composition of the infant gut after weaning.

Main results	Infant age	Weaning age	Type of solid foods	Sequencing method	Ref
The transition from breastfeeding to introduction of solid foods in infants may be a driver of metabolic changes in the fecal microbiota	Days 103−108 and 145−147, Days 165−175, Days 219−228 and 262−268	Start to complement food (days 165−175). Fully weaning (days 219−228 and 262−268)	From the seventh month, the infant consumed two semi-solid meals including meat (lamb, beef), fish (sole, sea bass), vegetables, eggs, ricotta cheese, and fruits.	Shotgun	[36]
A rapid and permanent shift in microbial composition has been observed within 5 d after the transition from human milk to cow’s milk.	5.5–17 months	4 months	Oatmeal, fruits, yogurt, and proteins such as tofu, eggs, and meat.	16S rRNA gene sequencing	[31]
The introduction of solid foods caused a sustained increase in the abundance of *Bacteroidetes*	Days 172−297-Days 454−838	Without mentioning the exact weaning time	Formula and peas	16S rRNA gene sequencing	[37]
The gut microbiota develops progressively, and its maturation pattern is closely associated with the development of systemic acid-base metabolism.	1 month, three months, 6 months, 12 months, 24 months, and 36 months	Weaned at 5 months (*n* = 3), 6 months (*n* = 6), 10 months (*n* = 1)	No mention	16S rRNA gene sequencing	[38]
The establishment of enterotypes occurs between 9 and 36 months of age, with approximately 30% of individuals undergoing an enterotype shift between 18 and 36 months. The timing of breastfeeding cessation has the most significant impact on the composition of the gut microbiota.	9−18 months	9−18 months	No mention	qPCR on Maxwell 16	[39]
A distinct evolutionary clade of *Bifidobacterium longum* has been identified, which expands following the introduction of solid foods in conjunction with continued breastfeeding.	15 months, 18 months and 24 months	No mention	No mention	16S rRNA gene sequencing and qPCR	[40]
The effects of human breast milk feeding on the infant gut microbiome are independent of the introduction of solid foods.	From 3 months to 6 months, samples were collected every month. From 7 months to 10 y, samples were collected every 3 months.	No mention	No mention	16S rRNA gene sequencing and shotgun	[41]
*B. infantis* dropped from 23.7% to 3.2% after weaning	3, 6, 12, 18, 24 and 36 months	No mention	No mention	16S rRNA gene sequencing Shotgun	[42]

While several studies have examined how the composition of the infant gut microbiota changes after weaning, the samples in these cohort studies were taken at various times, which affected the conclusions drawn about microbial alterations.^31^^,^^36^^,^^43^ Alongside the shift from a breast milk-based diet to a weaning diet, a typical modification in microbiota composition has been observed. Infants aged 220–268 days, transitioning from a diet mainly based on breast milk to weaning, showed a high relative abundance of *Ruminococcus*, *Lachnospiraceae*, and unclassified *Ruminococcaceae*.^36^ A study of the infant gut microbiota from 5.5 to 17 months of age, before cessation of human milk feeding, revealed that the *Bacteroidota* to *Bacillota* ratio began to increase. The relative abundances of *Bacteroides* spp., *Blautia* spp., *Parabacteroides* spp., *Coprococcus* spp., *Ruminococcus* spp., and *Oscillospira* spp. showed a significant increase in relative abundance at the genus level after weaning, whereas the relative abundance of *Bifidobacterium* spp., *Lactobacillus* spp., and *Escherichia* spp. declined.^31^ Most of these studies used discrete time-point sampling without defining the duration or type of food consumed, or the overlap between breastfeeding and supplemental feeding. Because of this temporal ambiguity, it is difficult to make strong inferences about causality, the order of microbial succession, or the generalizability of observed patterns. Attempts to identify "core weaning taxa" or universal microbial trajectories during this transitional period are hampered by the lack of longitudinal time-gradient analyses. To better describe the microbial landscape of weaning, future studies should prioritize standardized dietary documentation, including detailed nutritional data on food intake, along with dense, time-resolved sampling.

### Microbiota composition of the infant gut beyond 12 months

The early gut microbiota undergoes a transition from lactose-utilizing microbes during exclusive breastfeeding to anaerobic organisms that begin digesting solid foods, including complex carbohydrates, after their introduction.^37^ The infant gut microbiota develops into a more complex structure during the first year of life and reaches an adult-like composition by the age of three years.^3^ Several cohort studies have documented these gradual shifts, yet limitations in study design often obscure precise attribution of microbial changes to specific dietary or temporal factors. A cohort study from St. Louis revealed that the introduction of solid foods caused an increase in the relative abundance of Bacteroidota (days 172−297) that lasted until days 454−838, but the study did not specify when solid foods were introduced or the feeding method before weaning.^37^ Another study focused on healthy Japanese infants within the first 3 y of life that suggested an increase in the relative abundance of *Lachnospiraceae* and *Ruminococcus* during weaning, without differentiating food types.^38^ In a 3-y Danish study including 330 infants, the authors found that when solid foods were introduced to infants between the ages of 9 and 18 months, the bacterial composition of the gut microbiota changed significantly, from being dominated by *Bifidobacterium*, *Lactobacillus*, and *Enterobacteriaceae* to a microbiota dominated by *Clostridium* spp. and *Bacteroides* spp.^39^ Only one study has examined the structure of the infant gut microbiota at the species level following weaning. In that study, after the introduction of solid foods, the relative abundance of *B. longum* subsp. *longum* increased throughout the second year of life, replacing the dominant *B. longum* subsp. *infantis* during the early stages of infancy.^40^ This study provides an isolated example of species-level analysis but lacks comprehensive research that combines longitudinal sampling with dietary metadata. Therefore, the species-level composition of the infant gut microbiota throughout the weaning process requires further investigation. The field needs a detailed mechanistic understanding of how different taxonomic groups change in response to dietary shifts and host developmental stages. Addressing this gap will require species- and strain-level metagenomics to study both taxonomic details and functional changes during the second and third years of life.

### The metabolites of gut microbes shift during weaning time

#### Short-chain fatty acids

Short-chain fatty acids (SCFAs) are the main metabolites produced by the gut microbiota,^44^ which are closely linked to gut homeostasis and immunity.^45^ Increased microbial colonization and SCFA production are thought to trigger a strong immune response during the transition from breastfeeding to solid foods, a phenomenon known as the “weaning response” that has not been rigorously demonstrated in humans.^46^ The available data suggest that SCFA production increases significantly during this phase (Figure 1B).^47^ For example, Conta et al. observed a significant increase in SCFAs (acetate, propionate, butyrate) and ethanol during the weaning phase. This increase was associated with higher levels of amino acids and their intermediates, which are involved in protein fermentation (such as *β*-alanine and 4-hydroxyphenylalanine) and catabolism (branched SCFAs).^36^ After weaning, complex plant polysaccharides and resistant starches, which are otherwise indigestible, are broken down by SCFA-producing bacteria in the infant gut. Notably, the limited ability of pre-weaning microbial communities to metabolize complex carbohydrates may create an opportunity for exogenous or low-abundance microbes to utilize these nutrients, facilitating their successful establishment during the weaning process.^48^ For example, the relative abundance of members of the family *Muribaculaceae* (formerly known as s24−7) increased from 2% pre-weaning (day 14) to 36% (day 21) and then stabilized at 50%, as demonstrated in a mouse model. *Muribaculaceae* contains glycoside hydrolases capable of breaking down host-indigestible starches such as plant cell wall components, arabinan, xylan, and pectin.^49^ As solid foods are introduced, new and diverse species begin to appear.^39^ For example, *Roseburia*, *Faecalibacterium*, the *Clostridium leptum* group and *Ruminococcus* exhibit digestive abilities that allow them to survive in environments rich in non-digestible dietary fiber. These species can also produce butyrate, a key metabolite linked to the control and balance of the gut microbiota.^37^^,^^50^ However, a study found that the levels of butyrate-producing bacteria (*C. leptum* group, *C. coccoides* group, *E. hallii*, and *Roseburia* spp.) in infants decreased when weaning occurred later than 9 months of age.^39^ Supporting this, McKeen et al. reported that infants who began weaning later (at 9 or 12 months) exhibited lower microbial gene annotations related to carbohydrate, amino acid, and protein metabolism compared to those weaned at around 4 months.^51^ Studies have not determined the mechanism that aberrant microbial function occurs if weaning is delayed. The weaning response must resolve appropriately during ontogeny to prevent intestinal barrier dysfunction and long-term inflammatory response control abnormalities.^46^ This critical period of microbiota growth requires additional longitudinal studies to understand how timing, nutrient intake, and host‒microbe interactions influence SCFA trajectories.

#### Bile acids

In addition to their traditional role in lipid digestion, bile acids are also acknowledged as signaling compounds that actively interact with the gut microbiota, influencing the host immune system, intestinal barrier integrity, and the microbial community composition.^52-55^ Across different life stages, significant changes occur in bile acid metabolism as both the ability to synthesize bile acids and the microbiota's ability to transform them mature – an evolution that appears closely tied to the introduction of solid foods (Figure 1B).

The host initially produces conjugated bile acids, which are later transformed by the gut microbiota into deconjugated primary and secondary bile acids that affect varrious host systems. The liver produces bile acids (cholic acid and chenodeoxycholic acid), which then combine with glycine or taurine to form different bile acids such as deoxycholic acid (DCA), lithocholic acid (LCA), and ursodeoxycholic acid (UDCA).^56^ The newborn gut lacks the necessary bacteria to process bile acids, which therefore remain mostly unchanged after birth.^57^ The continuous colonization of different microorganisms occurs with increasing time after birth, and the introduction of solid food during weaning leads to intestinal bacteria with the ability to transform primary bile acids into secondary bile acids.^58^ For example, *Bacteroides*, *Clostridium*, *Escherichia*, *Egghertella*, *Eubacterium*, *Peptostreptococcus*, and *Ruminococcus* oxidize and epimerize bile acids, most likely contributing to the transitory surge in the levels of UDCA and iso-bile acids.^59^ One study reported an increase in epimerized bile acids, such as UDCA between 6 and 24 months of age, ^38^ while some studies reported secondary bile acids as early as 2–3 months of age, whereas others not until up to 24 months of age, depending on the time of weaning and the introduction of solid foods.^60^ Bile acids facilitate the maturation of the infant intestinal microbiome, with their concentrations exhibiting age-dependent variations. Hepatic concentrations of bacterially modified secondary bile acids increase with age, whereas the concentrations of primary bile acids decrease, as observed in a mouse model.^61^ However, the bile acid composition differs between humans and mice, so this finding may not fully reflect the range of human responses. A significant point to note is that much of the existing data are based on profiles of bile acids, which mainly capture metabolites from deconjugation and distal processes.^38^ This approach restricts our understanding concerning the cycle and the pool of bile acids in proximal regions of the small intestine, where absorption and initial microbial transformations occur. The regulatory function of bile acids during the weaning process has not yet been thoroughly investigated. There remains uncertainty about whether bile acid changes play an active role in shaping development during weaning or simply mirror the metabolic abilities of the microbes. Future research should combine the analysis of bile acids with the sequencing of the microbiome in populations over time to better understand how the interactions between bile acids and gut bacteria influence the health of individuals during infancy and early childhood.

#### Tryptophan

The intestinal microbial tryptophanase breaks down tryptophan to produce indole, which controls various host–microbiome homeostasis processes through its derivatives.^62^ Tryptophan undergoes three different metabolic pathways within the host body: the kynurenine pathway, the serotonin (5-HT) pathway, and the indole pathway (occurs in the gut microbiota).^63^ The weaning transition leads to major shifts in gut microbiota composition, which modify tryptophan metabolic pathways. Microbes involve enzymatic pathways that break down tryptophan into beneficial indole derivatives, including indole-3-lactic acid (ILA), indole-3-acetic acid (IAA), and indole-3-propionic acid (IPA) (Figure 1C).^63^^,^^64^ Microbial tryptophan metabolites have been demonstrated to improve intestinal barrier function, protect against pathogenic organisms, and affect host metabolism through interactions with the aryl hydrocarbon receptor (AhR) or pregnane X receptor (PXR).^44^^,^^64-67^ Research on tryptophan metabolic changes during weaning remains scarce, particularly in human infant populations. Tryptophan and its metabolites have been reported to be prematurely elevated in infants who are weaned early such as those weaned before 3 months, and in infants with asthma (Figure 1C).^68^ The gut microbiota also produces tryptamine and indole metabolites that activate the AhR receptor to control asthma through their regulation of innate and adaptive immune responses, as well as response to environmental and dietary molecules.^69-71^ Further research is necessary to understand the function of tryptophan and its metabolites during this developmental period because current knowledge about these topics remains scarce.

### Factors affecting the gut microbiota of infants through the weaning transition

The evaluation of weaning effects is challenging because of multiple variables, including feeding approaches (human breastmilk, formula milk, and mixed feeding) and factors such as gestational age, delivery mode, antibiotic use, geographical location, and the timing of solid food introduction (Figure 1C). Understanding the impact of these factors will help promote optimal infant nutrition and healthy gut microbiome development during this essential developmental period.

#### Feeding method before weaning

The feeding methods of breastfeeding, formula milk, and mixed feeding determine the structure of the infant gut microbiota.^72^ The advantages of breastfeeding for mothers include lower rates of hypertension, hyperlipidemia, and cardiovascular disease.^73^ Human breast milk contains sugars, fats, proteins, and micronutrients that significantly benefit infant health by supporting neural development, enhancing immune function, promoting intestinal barrier maturation, and facilitating bone growth.^74^^,^^75^ Infants fed exclusively with human breast milk harbor more *Bifidobacterium* in the gut compared to those fed non-exclusively, primarily due to the presence of HMOs.^76^ HMOs, the third most prevalent component in breast milk after lipids and proteins, are indigestible to infants but are preferentially utilized by gut microbes.^77^ Infant-type *Bifidobacterium* species, including *B. longum* subsp. *infantis*, *B. breve*, and *B. bifidum* possess GH family gene clusters that enable them to effectively absorb and process HMOs.^78^^,^^79^ HMOs are absent in conventional formulas, but some modern formulas now contain synthetic HMOs such as 2′-fucosyllactose (2′-FL), 3-fucosyllactose (3-FL), lacto-*N*-tetraose (LNT), 3′-sialyllactose (3′-SL), and 6′-sialyllactose (6′-SL).^80^ Yet, the complexity and structural diversity of natural HMOs surpass those of synthetic HMOs, which might restrict formula's ability to replicate the microbiota-shaping effects of human milk. Emerging evidence indicates that these impacts continue long after the infant has been weaned,^81^ despite the fact that the introduction of solid food drives these changes.^5^ In addition, the dynamics of bacterial composition in infants depend on the volume of human breast milk consumed until weaning.^82^

The introduction of solid foods does not determine when breastfeeding stops. Some studies have reported that changes in the intestinal microbiota caused by solid food introduction are linked to the timing of breastfeeding cessation.^83^ Other studies suggest that the cessation of breastfeeding has a greater influence on the infant gut microbiota composition than the introduction of solid food, transitioning the microbiota structure toward an “adult-like” state.^3^^,^^84^^,^^85^ This mature microbial profile, which is predominantly dominated by *Bacteroidota* and *Bacillota,*^3^^,^^86^ is more resistant to environmental stresses.^87^ Independent of the introduction of solid foods, breastfeeding cessation has a significant influence on gut microbiota composition.^41^ Bäckhed et al. found that, compared to infants fed mixed foods, exclusive breastfeeding was linked to reduced relative abundances of *Bacteroides*, as well as less phylogenetic diversity from birth to 12 months of age.^5^ When solid food is added to breast milk and formula feeding throughout the weaning process, some of these variations can persist.^88^ For example, breastfed infants showed higher relative abundances of *Bifidobacterium* and a lower relative abundances of *Bacteroides* compared to those who were fed formula during the same time period.^32^ Thompson et al. found variations in the microbiota of infants who were exclusively breastfed compared to those who were not, both before and after the introduction of solid foods.^89^ Solid foods are first introduced to breastfed infants and are associated with the appearance of *Veillonella*, *Roseburia*, and members of the *Lachnospiraceae* family; nevertheless, infants who were not exclusively breastfed harbored *Streptococcus* and *Coprobacillus* after consuming solid food.^90^ These findings strongly suggest that feeding methods before weaning significantly influence the infant’s gut microbiota, even after weaning. However, the question of whether it is appropriate to make separate weaning recommendations for breast-fed and formula-fed infants has yet to be considered.

#### Gestational age

Gestational age is a critical factor influencing the infant gut microbiota composition after birth.^91^ Preterm infants face greater risks of growth restriction and altered body composition when they reach term-equivalent ages compared to full-term babies.^92^ The introduction of solid foods to preterm infants during their immature gut state creates a greater risk of disrupting normal gut microbiota development.^93^ Therefore, researchers have suggested that preterm infants should have different feeding schedules because their recommendations differ from one another.^94^ For example, Gupta et al. recommend starting solid food introduction at 6 months for infants born preterm before 34 weeks of gestation.^95^ In contrast, Palmer and Makrides recommend starting solid food introduction for preterm infants at 3 months (13 weeks) of corrected age.^94^ These differing guidelines demonstrate that there is insufficient high-quality microbiota-based evidence to establish weaning protocols for preterm infants. Research indicates that weaning strategies should take gestational age into account, because premature solid food introduction during immature physiological states may harm gut microbiota development and future health outcomes.

#### Delivery mode

Substantial evidence indicates that the structure of the gut microbiota is altered by cesarean section birth^96^ up to the age of 2 y^97^, 4 y^98^ or 7 y^99^ based on different studies. However, the lack of specific information about weaning timing, dietary patterns, and nutrient intake in existing studies makes it difficult to determine how solid food introduction affects gut microbiota development based on birth mode. The exact timing of gut microbiota changes during weaning in infants born via C-section versus vaginal delivery remains unknown, as does the relative impact of delivery method versus weaning practices. Future research should focus on delivering studies that divide participants based on birth mode while collecting frequent microbiota samples during weaning and maintaining complete records of dietary information. The combination of metagenomics with metabolomics and host immune profiling through multi-omics approaches will help determine how the delivery mode affects weaning-induced microbial and metabolic changes and whether specific nutritional interventions can reduce long-term health risks for infants born via C-section.

### The use of antibiotics before weaning

The application of broad-spectrum antibiotics has proven to be life-saving for infants suffering from different diseases,^100^ but misuse during prenatal and postnatal periods can promote antimicrobial resistance in the gut microbiota.^101^ The maternal gut and breast milk contain antibiotic-resistant bacteria, which develop from increased antibiotic consumption during pregnancy and breastfeeding before transferring to the infant's gut.^102^^,^^103^ The genetic barcode analysis revealed that five infant‒mother pairs shared highly similar antibiotic-resistant *Bifidobacterium longum*, *Limosilactobacillus fermentum*, *Lactobacillus gasseri,* and *Enterococcus faecalis.*^104^ Research conducted in Taiwan demonstrated that multi-resistant bacterial strains present in human breast milk can pass to infants.^105^ The long-term health effects of antibiotic exposure and antimicrobial resistance in infants remain uncertain, particularly after they stop breastfeeding. A Danish infant cohort study demonstrated that antibiotic exposure during cord clamping resulted in the detection of more microbial species in infants at 9 months of age.^106^ Since the authors expected the effects of antibiotic exposure to become more noticeable at 10 d, they concluded that although antibiotic exposure may have played a role in the differences observed at 9 months of age, it was more likely the result of the intestinal microbiota reorganizing after weaning.^106^ The research by Fouhy et al. revealed no changes in infant gut microbiota during their first year of life after antibiotic treatment.^98^ The infants in this study received antibiotics at different points between 3 weeks and 12 months of age. The different durations of antibiotic exposure create challenges for drawing general conclusions. The growing body of evidence demonstrates the necessity for specific investigations of antibiotic and antimicrobial effects on the infant gut microbiota especially during the weaning and post-weaning periods.

#### Geographical location

As with other microbiome studies, the structure of bacterial compositional changes after weaning varies by country.^32^^,^^107^ A cohort from Italy indicated that the variation in *Bifidobacterium* abundance appeared to occur independently of the feeding transition, based on an analyses of infant gut microbiota at the age of 4–10 months, and these infants were exclusively fed breast milk.^36^ Studies from St. Louis (USA) and European countries (Sweden, Scotland, Germany, Italy, and Spain) suggest that *Bifidobacterium* (36.5%), *Clostridium coccoides* (14%), and *Bacteroides* (13.6%) are dominant in the gut of breastfed infants after weaning.^32^^,^^37^^,^^108^ Other studies also found that *Bacteroides* emerged as a predominant genus with the introduction of solid foods,^109^ accompanied by *Alistipes*, *Dialister*, *Prevotella*, *Faecalibacterium*, *Ruminococcus*, *Roseburia*, *Eubacterium*, and *Akkermansia*; however, these studies did not differentiate the feeding methods.^109-111^ In addition, the relative abundance of *Atopobium*, *Clostridium*, *Akkermansia*, *Bacteroides*, *Lachnospiraceae*, and *Ruminococcus* species increased, while *Escherichia* and *Staphylococcus* species decreased during the weaning period.^107^ However, one study found that *Prevotella* was the main bacterium in the infant gut after weaning based on a cohort from Indonesia.^112^^,^^113^ These observed differences stem from local dietary patterns (e.g. high-fiber plant-based diets), early-life environmental microbial exposures and host genetic factors. The majority of current research uses observational methods while showing significant variability in their sampling periods and their classification of feeding practices and documentation of weaning. Future research should establish cross-cohort comparative frameworks to control for feeding mode, dietary content, and weaning timing. Furthermore, the distinction between compositional variability and functional convergence or divergence across regions will require integrating functional data, including metagenomic, metabolomic, and dietary profiling.

#### The timing of weaning

The development of the infant gut microbiota is divided into several stages based on different studies.^41^^,^^114^ Weaning time varies along with the development of the infant gut microbiota and typically begins around 4−6 months of age and lasts until the child reaches 2 y old.^3^ Although the WHO recommends that infants are breastfed until 6 months, studies have shown that some infants are weaned before 6 months of age.^33^ The Infant Feeding Survey in the United Kingdom found that 81% of women began breastfeeding after birth, yet the breastfeeding continuation rates at 1 week, 6 weeks, and 6 months decreased to 69%, 55%, and 34%, respectively. ^115^ The survey showed that 34% of mothers breastfed exclusively for 6 months, yet 30% started solid foods before 4 months and 75% before 5 months.^115^ The introduction of solid foods prior to 4 months triggered rapid changes in infant gut microbiota development.^82^ The premature change in the gut microbiota composition leads to increased obesity risk during adulthood^116^ because it allows bacteria to become more efficient at food energy extraction, which results in better energy absorption and fat storage.^117^ The correct timing of weaning determines the maintenance of gut microbiota equilibrium, which leads to long-term wellness.

Research has been conducted on the time at which breastfeeding starts and when solid foods should be introduced. For example, the Prevention and incidence of asthma and mite allergy (PIAMA) cohort in the Netherlands provides evidence that a brief period of breastfeeding (four months or less) is linked to a higher risk of childhood obesity, which is more important than the early introduction of solid foods. In contrast, no discernible difference in risk was found between infants who are breastfed and those who are formula-fed.^116^ This study, however, does not address the types of solid foods introduced, the overlap between breastfeeding and solid feeding, or potential microbial functional changes, such as mammalian target of rapamycin (mTOR), AMP-activated protein kinase (AMPK), and the insulin signaling pathway associated with obesity, that may be predicted by KEGG annotation based on shotgun analysis.^116^^,^^118^^,^^119^ In a different study, infants who received solid foods early (at 4 or 5 months) had a distinct gut microbiota compared with infants who started solids later,^120^ and some beneficial bacteria (e.g. *Bifidobacterium*) declined quickly if infants were weaned before 6 months.^121^ Similar to previous studies, the length of exclusive breastfeeding rather than the age at which infants are introduced to solid foods has been linked to reported improvements.^120^ A study reported that early weaning (before 3 months) can accelerate the colonization pattern of the microbiome, leading to the premature acquisition of specific microbial species and functions, including *Ruminococcus* and tryptophan biosynthesis, which increases the risk of asthma.^68^ Thus, weaning either too early or too late can have equally detrimental effects on the gut microbiome composition and immune system development.^122^ Although current research shows that the timing of solid food introduction does not significantly affect the development of the infant intestinal microbiota, premature weaning may increase the risk of diseases such as acute respiratory infections later in life.^123^ When infants at 6 months of age are given their first supplementary foods, the gut microbiome production of metabolites and beta diversity both rapidly change.^22^^,^^124^^,^^125^ These findings highlight weaning/introduction of solid foods as a critical driver of microbiome development, though the most appropriate timing remains to be fully established.

#### Type of diet used during weaning

Infants receive human breast milk or infant formula after birth and are eventually weaned onto a variety of foods,^126^ with the composition of the diet having a significant influence on shaping the gut microbiota. When the baby first starts to be weaned, a sizable number of starches (such as rice), plant-rich foods (such as vegetable broth), animal proteins (such as lamb baby food), and fibers are consumed. Weaning foods high in carbohydrates, such as fruits, vegetables, and cereals, partially replace higher-fat formula and breast milk, causing energy intake to decrease from around 50% of total energy during breastfeeding to 30%−35% of total energy.^37^ The termination of milk feeding/nursing leads to a rapid shift in infant gut microbiota and metabolites, which develop into an adult-like pattern with diverse and mature bacterial communities containing Bacillota and starch and protein degradation metabolic pathways according to 16S rDNA sequencing and metabolomics analysis.^36^ The pancreas of infants remains underdeveloped, so they cannot produce sufficient alkaline bicarbonate and digestive enzymes to neutralize stomach acid and break down sugars, proteins and fats in the intestine.^127^ The undigested nutrients that reach the colon serve as substrates that enable specific bacterial communities to grow and dominate.^128^ This may relate to the transition stage from an exclusive breast milk-based diet to the introduction of solid foods.

Introduction of different solid foods may lead to different microbial changes.^23^ For example, an *in vitro* study from Australia indicated that digested rice samples led to a considerably greater relative abundance of *Bifidobacteriaceae* than those containing digested oats, sorghum, or wheat. Additionally, the inclusion of digested oats was associated with a rise in *Veillonellaceae*.^129^ The ratio of whole grains to sugars also caused differences in the infant gut microbiota after weaning. Infants who received cereals containing 0% whole grains and 24 g/100 g sugars (0-WG) showed a decrease in the relative abundance of *Bifidobacterium*, while those given 50% whole grains and 12 g/100 g sugars (50-WG) showed an increased relative abundance of *Lachnoclostridium* and *Bacteroides*. Additionally, the relative abundance of *Pseudomonadota* and *Escherichia* was lower in the 50-WG group compared to that in the 0-WG group. These results are based on a study of a cohort of 4−7-month-old Spanish infants who received different ratios of whole grains and sugar in their first weaning foods over 7 weeks of intervention.^130^ Research has demonstrated that higher alpha diversity levels (observed species and Shannon index) were linked to both elevated fiber intake and diverse eating habits^22^ and high daily dietary diversity maintained gut microbiota stability throughout the research period, as observed in adult studies.^131^ Future research needs to conduct systematic assessments of how various food types and the timing of their introduction affect the infant gut microbiota after weaning to determine optimal dietary patterns for a smooth transition from breast milk or formula feeding.

### The developing functions of infant gut microbiome in response to weaning time

**Figure 2.**
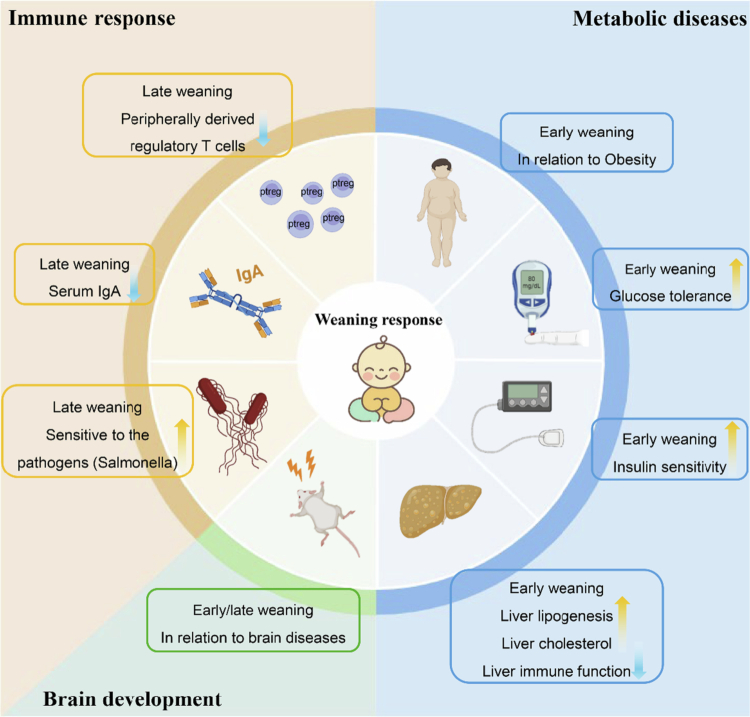
The changes in microbial functional fluctuation in the infant gut after weaning and related to host disease in response to weaning time. Microbial functions caused by different weaning times (early and late) may contribute to brain development, metabolic disease, and the immune response.

The developing functions of the infant gut microbiome in response to weaning time play crucial roles in shaping long-term health outcomes. Research on weaning effects on microbiome composition has been conducted, but the complete understanding of how these microbial changes affect host physiology and disease susceptibility remains unclear. Additional research is required to understand the complex relationship between weaning duration and microbiota development and long-term health outcomes while emphasizing the necessity of organized studies during this essential period of early-life gut microbiota development (Figure 2).

#### Brain development

The microbiome establishes connections between the brain through the gut‒brain axis, which affects both brain development and behavioral processes.^132^ In a study by Rothenberg et al. 3-y-old children were studied to identify microbial factors that showed positive coabundances of *Faecalibacterium*, *Clostridium cluster XIVa*, *Gemmiger*, *Phascolarctobacterium*, *Alistipes*, *Oscillibacter,* and *Sutterella* alongside negative coabundances of *Blautia*, *Anaerostipes*, *Clostridium* cluster XVIII, and *Streptococcus*. The microbial profile showed positive links to both mental and psychomotor development according to the BSID (2nd edition) assessment.^133^^,^^134^ Weaning opens an optimal window for infants to gain an extra microbiome that may influence brain development.^132^^,^^135^^,^^136^ However, weaning time is considered a critical factor contributing to brain development. A study revealed that delaying the weaning period may contribute to the development of depression in a rat model, which is associated with an increased level of *N*-acetylglycoprotein, a recognized biomarker of depressive disorders.^137^^,^^138^ Additionally, early weaning has been shown to induce anxiety in mice, potentially through alterations in brain-derived neurotrophic factor (BDNF) signaling pathways.^139^ The composition of the microbiota influences the BDNF signaling pathway through the brain‒gut axis, thereby impacting brain development as well as the onset and progression of brain-related diseases.^140^ The research lacks direct measurement of microbiota composition and functional changes during weaning and specific effects of dietary components on microbial neuroactivity is lacking. This phenomenon has been observed only in animal models. Future research needs to combine human cohort studies with behavioral assessments and microbiome and neuroimaging or neurodevelopmental metrics to bridge this knowledge gap. Research on how weaning timing and diet affect microbiota–central nervous system interactions could lead to new nutritional approaches for early-life neurodevelopment and mental health optimization.

#### Metabolic diseases

Metabolic disorders refer to a group of diseases or pathological conditions caused by abnormalities in the body's metabolic processes, including obesity, type 2 diabetes, metabolic dysfunction-associated steatohepatitis (MASH), and cardiovascular conditions,^141^ and there is an association between the microbiome, diet and disease development.^142^ The association between the timing of solid food introduction and childhood overweight/obesity has been reported in numerous experimental and epidemiological studies.^143^^,^^144^ Some articles have suggested that weaning that starts at 3 and 4 months of age increases the risk of obesity during childhood, according to a cohort of African Americans.^145^ Rat models also demonstrated that early weaning resulted in increased glucose tolerance, insulin sensitivity, liver lipogenesis and liver cholesterol, reduced liver immune function; and accelerated metabolic function transitions during neonatal development.^146-148^ Early weaning negatively impacts liver metabolic enzymes, which may be due to the altered structure of the gut microbiome, as demonstrated by piglet models.^149^ Therefore, investigating the role of the gut microbiome as it undergoes reprogramming during the weaning period can provide valuable insights into the significance of proper weaning timing.

#### Immune response

The crosstalk between the gut microbiome and immunity starts at birth.^27^^,^^150^ Asthma, food allergies and atopic dermatitis represent the most widespread immune diseases that develop after microbiome exposure to the body.^151^ Th2 and Th1 responses maintain a continuous balance that leads to allergic diseases as a result of their imbalance.^152^ The Th2 response that children inherit from their mothers occurs at birth until they reach the Th1/Th2 balance, which scientists have not yet determined.^27^ The development of immunity strongly depends on the gut microbiome during the early stages of life.^153^ Infants delivered through cesarean section develop microbial communities that differ from those of vaginal births in both structure and timing and present reduced microbial diversity and delayed anaerobic bacteria colonization, including *Bifidobacterium* and *Lactobacillus*, and a higher prevalence of skin and hospital environment microbes, such as *Staphylococcus*, *Corynebacterium*, *Propionibacterium* spp., *Enterococcus*, *Enterobacter* and *Klebsiella* species.^96^^,^^154-156^ The early-life microbial patterns have been linked to increased susceptibility to allergic conditions.^157^ The immune system development is delayed by the restriction of commensal species present before weaning because it leads to decreased peripheral regulatory T (pTreg) cells in the intestine along with low serum IgA levels and increased pathogen sensitivity (*Salmonella*) in a mouse model.^48^ A gnotobiotic mouse model demonstrated how certain commensal microorganisms produce IgA to affect immune system functions,^158^^,^^159^ pTregs and innate lymphoid cells. pTregs are Treg cell subtypes that control inflammation to maintain immunological tolerance while blocking autoimmune disorders and IgA plays a role in mucosal immunity through its anti-inflammatory properties and immune system regulation.^160^^,^^161^ The gut microbiome triggers the production of pTreg cells and IgA during weaning through the weaning response to ensure healthy immune system development and prevent future immune system pathologies.^46^ However, these findings should be interpreted with caution. Most experimental studies have been conducted in animal models, which differ from humans in terms of developmental pace, microbiota composition, and weaning practices. Research using cohort studies has failed to provide detailed microbiome information, and the pathways through which these effects occur remain unclear. The weaning period requires both balanced microbiome restoration and strong immune system development.

#### Concluding remarks and future perspectives

The shift from milk-only nutrition through breastfeeding or formula feeding to solid food introduction strongly affects the development of infant gut microbiota, which directly influences overall infant health. The introduction of solid foods enables the infant gut microbiota to evolve from *Bifidobacterium* dominance toward an adult-like diverse microbial community. Multiple factors determine the microbial shift including feeding methods and gestational age, mode of delivery and antibiotics exposure and the timing of solid foods introduction, food types and geographical location. The modifications in the microbiome structure affect different aspects of infant health, including neural development, metabolic disorders, and the immune response.

The factors mentioned play a crucial role in shaping the infant gut microbiome, yet their specific effects on microbiome composition and function during weaning require further investigation. Additional research is needed to understand the mechanisms behind microbiota changes during weaning because this knowledge will help develop effective weaning strategies. The process of weaning creates changes in microbial composition while simultaneously affecting microbial functions, which may contribute to disease development. Future research should focus on exploring the functional diversity of the gut microbiome and its complex host relationships because this field remains poorly understood. Research on weaning-related functional changes will help us understand how this transition affects the infant gut microbiome (Table 2). The acquired knowledge will help us select appropriate weaning schedules and feeding options for infants, which will lead to improved long-term health outcomes.

**Table 2.** Future research directions: based on gut microbiome reprogramming during infant weaning.

Research direction	Description
Microbiota dynamics and keystone species	Longitudinal profiling of key microbial shifts from breastfeeding/formula to post-weaning stages
Factors influencing Microbiota	Comparative analysis of various factors shaping the gut microbiota
Vertical Transmission and Weaning Disruption	Tracking maternal microbial strains and their persistence or replacement during weaning or even into adulthood
Microbial Metabolites and Host Development	Investigation of SCFAs, tryptophan metabolites, and other microbial metabolites during microbiome reprogramming
Mechanistic study of Microbe-Host Interactions	Exploring how gut microbes influence brain development, immune responses, and metabolic diseases, investigating the relationship between microbiome changes during weaning and these host processes
